# Evaluation of two intraoperative gamma detectors for assessment of ^177^Lu activity concentration in vivo

**DOI:** 10.1186/s40658-016-0168-x

**Published:** 2017-01-09

**Authors:** Viktor Sandblom, Ingun Ståhl, Roger Olofsson Bagge, Eva Forssell-Aronsson

**Affiliations:** 1 Department of Radiation Physics, Institute of Clinical Sciences, Sahlgrenska Cancer Center, Sahlgrenska Academy, University of Gothenburg, SE-413 45 Gothenburg, Sweden; 2 Department of Surgery, Institute of Clinical Sciences, Sahlgrenska Cancer Center, Sahlgrenska Academy, University of Gothenburg, SE-413 45 Gothenburg, Sweden

**Keywords:** Intraoperative gamma detectors, Performance evaluation, ^177^Lu, Neuroendocrine tumours

## Abstract

**Background:**

Patients with somatostatin receptor-expressing neuroendocrine tumours can be treated with intravenously administered ^177^Lu-octreotate. Few patients are cured with the present protocol due to the current dose limitation of normal organs at risk, such as the kidneys. By locally administering ^177^Lu-octreotate to the liver for the purpose of treating liver metastases, a substantially reduced absorbed dose to organs at risk could be achieved. The development of such a technique requires the capability of measuring the ^177^Lu activity concentration in tissues in vivo. The aim of this study was to evaluate different performance parameters of two commercially available intraoperative gamma detectors in order to investigate whether intraoperative gamma detector measurements could be used to determine ^177^Lu activity concentration in vivo.

**Results:**

Measurements were made using different sources containing ^177^Lu. Response linearity, sensitivity, spatial resolution and its depth dependence, organ thickness dependence of the measured count rate and tumour detectability were assessed for two intraoperative gamma detectors. The two detectors (a scintillation and a semiconductor detector) showed differences in technical performance. For example, the sensitivity was higher for the scintillation detector, while the spatial resolution was better for the semiconductor detector. Regarding organ thickness dependence and tumour detectability, similar results were obtained for both detectors, and even relatively small simulated tumours of low tumour-to-background activity concentration ratios could be detected.

**Conclusions:**

Acceptable results were obtained for both detectors, although the semiconductor detector proved more advantageous for our purpose. The measurements demonstrated factors that must be corrected for, such as organ thickness or dead-time effects. Altogether, intraoperative gamma detector measurements could be used to determine ^177^Lu activity concentration in vivo.

## Background

Neuroendocrine tumours (NETs) originate in neuroendocrine glands and are often characterised by overexpression of hormone receptors and their ability to store and secrete peptides and neuroamines [1]. They are generally small and slow growing and their symptoms are often vague or even indiscernible [2]. Thus, NETs have often metastasised by the time of diagnosis and the possibility of cure by surgical removal of the primary tumour is unlikely. There are several different systemic treatment options for these patients, including the use of somatostatin analogues, chemotherapy, and targeted therapies including radionuclide therapy [3]. Specifically, treatment with the somatostatin analogue ^177^Lu-octreotate has shown promising results [4–11]. However, few patients achieve complete remission and there is a need for improvement of ^177^Lu-octreotate treatment, with some options being (1) increasing the therapeutic effect on tumour tissue or (2) reducing the uptake in healthy organs at risk [12]. By lowering the uptake in organs at risk, the administered activity, and hence the absorbed dose to the tumour, could be increased.

For systemic ^177^Lu-octreotate treatment, kidneys and bone marrow are the main organs at risk and therefore restrict the activity that can be administered to the patient without causing severe negative side effects due to high absorbed doses [5, 6, 8, 13–15]. By introducing a new treatment method, based on local administration of ^177^Lu-octreotate to the liver, where NET metastases often are localised, a substantially lower uptake in kidneys and bone marrow could be achieved. Using this treatment method, the liver would be the main organ at risk and the development and optimisation process would require detailed and accurate determination of ^177^Lu-octreotate concentration in the liver, but also in other tissues such as tumours and the kidneys, in vivo. Knowledge of time-dependent activity concentrations of ^177^Lu-octreotate is required for calculation of absorbed doses.

An intraoperative gamma detector is a small handheld instrument for gamma radiation measurements [16]. Since ^177^Lu emits both electrons and photons, it is possible that such a detector could be used to accurately quantify ^177^Lu concentration during a therapy situation. Normally, intraoperative gamma detectors are used for radioguided surgery, where the purpose is to locate and surgically remove small lesions after injection of a diagnostic radiopharmaceutical [17]. This method may offer clinical benefit for patients by minimising the invasiveness of the surgery or enabling more tumour tissue to be removed. Radioguided surgery is used for many different clinical conditions, one of which is NET using ^111^In-octreotide as the tracer. Several studies have demonstrated that intraoperative detectors can detect even relatively small tumours if properly used [18–22].

During the last few decades, many studies have evaluated the technical performance of intraoperative gamma detectors [23–35]. Most of these were performed for diagnostic radionuclides (e.g., ^99m^Tc or ^111^In). More recently, the possibility of using intraoperative detectors to locate small lesions in patients injected with therapeutic radionuclides, such as ^177^Lu and ^90^Y, has been investigated [36–38]. However, the technical performance of intraoperative gamma detectors when used for therapeutic radionuclides remains to be studied in more detail.

The aim of this study was to measure different performance parameters of two commercially available intraoperative gamma detectors in order to investigate the possibility of using intraoperative gamma detection for determining ^177^Lu activity concentration in small tissue regions in vivo.

## Methods

The performance of two intraoperative gamma detectors was assessed using different phantoms containing ^177^Lu (IDB Holland BV, Baarle-Nassau, the Netherlands). The parameters assessed in this study included response linearity, sensitivity, spatial resolution and its depth dependence, organ thickness dependence and tumour detectability. ^177^Lu activity was determined using a well-type ionisation chamber (CRC-15R, Capintec, Ramsey, NJ, USA).

### Gamma detectors

**Fig. 1:**
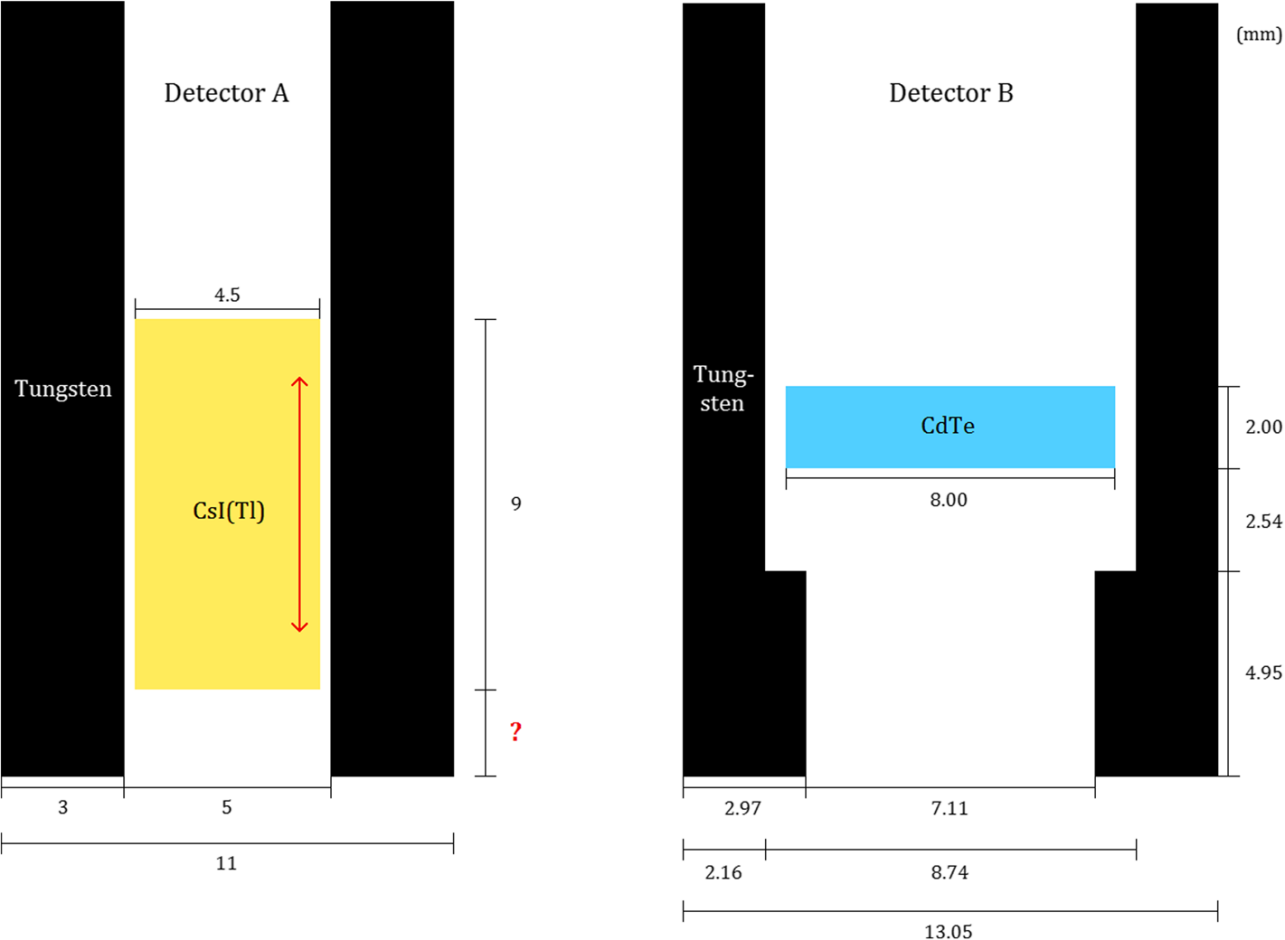
Schematic view of the geometrical crystal and collimator design of the two detectors. For detector A, information from the manufacturer about the distance from the crystal to the distal end of the collimator was not available, indicated by the *question mark* and the *arrow* inside the CsI(Tl) crystal. Note that only the tips of the two detectors are shown

The two intraoperative gamma detectors evaluated were the Gamma Finder II (World of Medicine GmbH, Berlin, Germany) and the Navigator GPS with the probe model “Standard lymphatic mapping” (Dilon Diagnostics, Newport News, VA, USA) (Fig. 1). The Gamma Finder II, hereafter called detector A, contains a scintillation detector with a CsI(Tl) crystal of 4.5 mm in diameter and with a height of 9 mm. The tungsten collimator has an aperture of 5 mm in diameter and a wall thickness of 3 mm. Detector A is wirelessly operated and the measured count rate is shown in counts per second (cps) in a display on the detector. The Navigator GPS, hereafter called detector B, contains a semiconductor detector. It has a 2-mm-thick octagonal CdTe crystal (with a dimension of 8 mm between two opposite corners) and a tungsten collimator with an aperture of 7.11 mm in diameter and a wall thickness of 2.97 mm. Detector B consists of a probe connected by a cable to a control unit, where the measured count rate is displayed in cps. Both detectors offer the capability of performing a 10-s measurement, during which the total number of counts is recorded. The reason why our study was limited to these two detectors was that those were only ones to which we had access.

The measureable photon energies from ^177^Lu are 113 and 208 keV, with an emission yield of 6.17 and 10.3%, respectively [39]. Detector A has a non-adjustable low-energy threshold of 110 keV, that is, all registered photons with energy above 110 keV will be counted. Detector B has a set of selectable non-adjustable energy windows designed for different diagnostic radionuclides (^99m^Tc, ^111^In, ^125^I and ^131^I). A 20% energy window centered over the ^99m^Tc energy peak at 141 keV (range 112–169 keV) was used for all detector B measurements.

### Linearity and sensitivity

The response linearity of the two detectors was investigated using point sources. Twenty-six point sources (0.19–29 MBq) were prepared by placing small drops of ^177^Lu at the bottom of 1.5-ml Eppendorf tubes. The detector was placed immediately in front of each point source. The measurement time was adjusted so that at least 1000 counts were collected. The sensitivity of the two detectors, given as count rate per unit activity of the point source, was calculated from the data obtained in the measurements of response linearity.

### Spatial resolution and its depth dependence

To determine the spatial resolution and its depth dependence, a line source was created by filling a narrow plastic tube (inner diameter 0.86 mm) with ^177^Lu. The activity concentration in the tube was 500 MBq/ml, corresponding to 0.29 MBq/cm tube length. This line source was placed at different depths, ranging from 0 to 80 mm, in a block phantom of tissue-equivalent plastic (polymethyl methacrylate). The detector was mounted in a holder and positioned immediately above the phantom surface. The detector was then moved stepwise laterally across and perpendicularly away from the line source from −70 to +70 mm, where the position 0 mm corresponds to the position where the detector was centered over the line source. For each position, the number of counts during 10 s was recorded, resulting in about 100–10,000 counts depending on the distance from the line source. The spatial resolution was determined as the full-width-at-half-maximum (FWHM) and as the full-width-at-tenth-maximum (FWTM) of the line profiles obtained.

### Organ thickness dependence

To assess the dependence of organ thickness, a gel phantom was created from 2% agarose gel blocks, containing a homogeneous distribution of ^177^Lu with an activity concentration of 23 kBq/ml. To create the gel blocks, a mixture of tap water and 2% agarose gel powder (Agarose A9539, Sigma-Aldrich Corporation, St. Louis, MO, USA) was heated in a microwave oven until the gel powder was completely dissolved. Thereafter, ^177^Lu was added to the solution under agitation to obtain a homogeneous activity distribution. Lastly, the radioactive gel mixture was poured into moulds for solidification. The dimension of each block was approximately 250 **×** 170 × 10 mm. These blocks were stacked on each other to obtain thicknesses between 10 and 150 mm. The detector was placed in a holder and positioned immediately above the surface and at the center of the block. The measurement time for each thickness was adjusted so that at least 1000 counts were collected. Before each measurement, the height of the stack of blocks was determined using a ruler, due to small differences in the exact dimensions among gel blocks.

### Tumour detectability

**Fig. 2:**
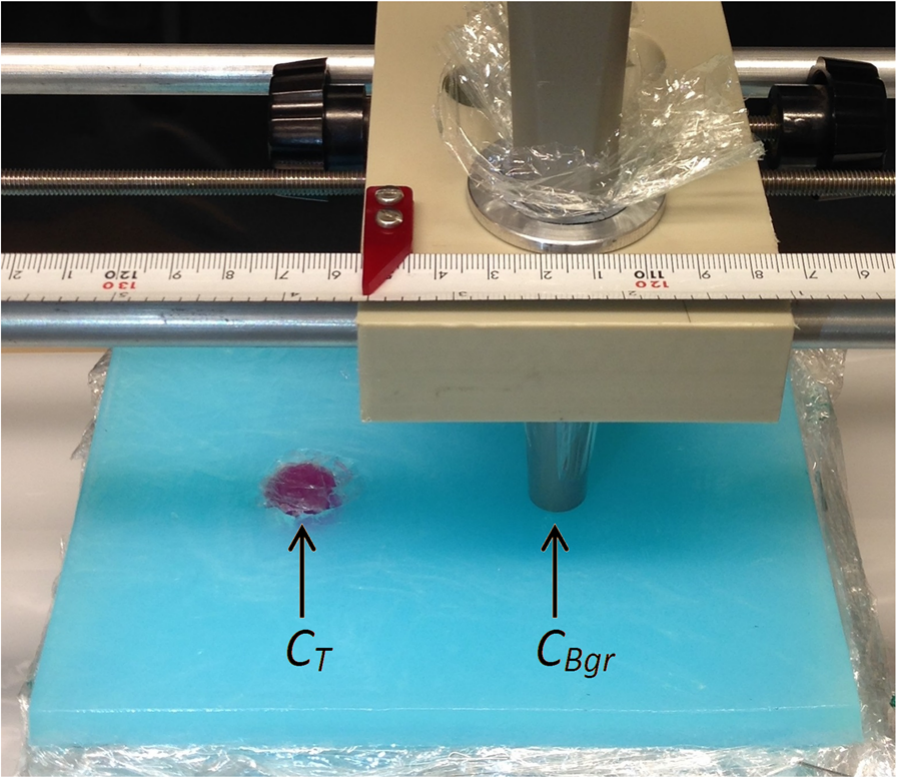
The positions in which *C*_*T*_ and *C*_Bgr_ was measured during the tumour detectability measurements. The red gel tumour sphere placed at the surface on the left simulated a tumour, and the blue background phantom simulated surrounding normal tissue

To investigate the tumour detectability of the two detectors, a tumour phantom was created by placing a 2% agarose gel sphere at the surface of a stack of 10 gel blocks (described earlier). The gel sphere was created by pouring radioactive gel mixture into a spherical mould. The gel sphere, simulating a small tumour, contained a higher activity concentration than the gel blocks, simulating the background, that is, the surrounding normal tissue. Measurements were made for different sphere sizes (diameter of 5, 10, 15 and 20 mm) and ratios between the activity concentration in the tumour sphere and that in the background (T/Bgr, 2, 5, 15 and 30). These sizes and ratios were chosen to be relevant for clinical situations [40–42]. The detector was mounted in a holder and positioned immediately above the surface of the stack of gel blocks. Measurements were made in two positions, (1) over the tumour sphere, where a number of counts, *C*_*T*_, was obtained and (2) over the background, where a number of counts, *C*_Bgr_, was obtained (Fig. 2). The measurement time for each position was adjusted so that at least 1000 counts were collected for each position. The tumour detectability measurements were carried out one day after the measurements of organ thickness dependence. Therefore, the activity concentration in the background gel blocks was 20 kBq/ml instead of 23 kBq/ml, due to radioactive decay.

The homogeneity of the activity concentrations in the tumour phantom was evaluated by measuring 16 random samples from the gel blocks and 10 random samples from the gel tumour spheres in a gamma counter (Wallac 1480 Wizard 3”, Wallac Oy, Turku, Finland).

### Statistical analyses

For the measurements of organ thickness dependence, statistical analyses were performed to determine the largest thickness where a statistically significant difference could be seen when the signal intensity at that thickness, C, was compared to the signal intensity measured at the largest thickness, *C*_Max_ (148 mm). Given a measurement time of 20 s, the standard deviation (SD) of the difference between *C*_Max_ and *C*, *σ*(*C*_Max_ − *C*), was calculated:

1 $$ \sigma \left({C}_{\mathrm{Max}}-C\right)=\sqrt{C_{\mathrm{Max}}+C} $$

Differences between *C*_Max_ and *C* were considered statistically significant (*p* < 0.05) when they exceeded two SDs of the difference, that is 2 × *σ*(*C*_Max_ − C).

For the tumour detectability measurements, statistical analyses were performed to determine for which T/Bgr and tumour sphere sizes the difference between *C*_*T*_ and *C*_Bgr_ was statistically significant, given the measurement time of 20 s. The SD of the difference between *C*_T_ and *C*_Bgr_, *σ*(*C*_*T*_ − *C*_Bgr_), was calculated:

2 $$ \sigma \left({C}_T-{C}_{\mathrm{Bgr}}\right)=\sqrt{C_T+{C}_{\mathrm{Bgr}}} $$

Differences between *C*_*T*_ and *C*_Bgr_ were considered statistically significant (*p* < 0.05) when they exceeded two SDs of the difference, that is 2 × *σ*(*C*_*T*_ − *C*_Bgr_).

## Results

### Linearity and sensitivity

**Fig. 3:**
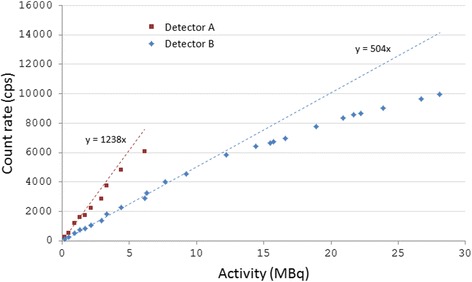
Detector response given as count rate for point sources of different radioactivity. The lowest 4 and 13 activity levels were used to plot the trend line as linear response for detectors A and B, respectively

The response increased when the activity of the point source increased (Fig. 3). The two detectors showed great differences in response. A linear response (±10%) was seen for activities up to 1.3 and 12 MBq for detectors A and B, respectively. Both detectors reached a maximum measurable activity. This maximum was about 5 times higher for detector B (28 MBq) than that of detector A (6.1 MBq). The sensitivity was higher for detector A than for detector B—1200 and 500 cps/MBq, respectively (Fig. 3).

### Spatial resolution and its depth dependence

**Fig. 4:**
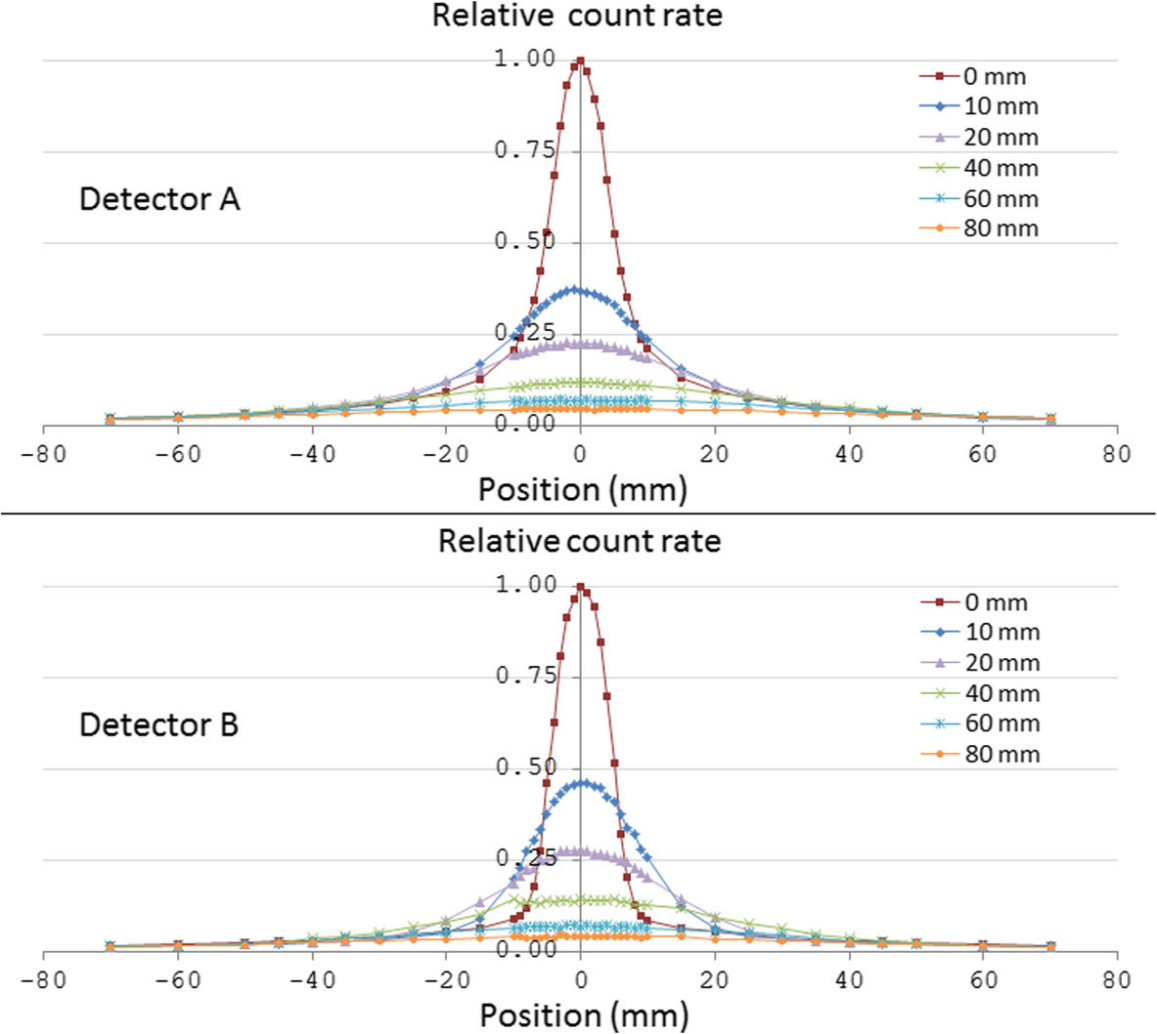
Detector response of the two detectors for the ^177^Lu line source located at a depth of 0–80 mm. The y-axes show relative count rate, i.e. signal intensity values normalised to the signal intensity measured at lateral position 0 mm at a depth of 0 mm. The uncertainty in the position of the detector was about ±0.5 mm for each measurement

The line profiles acquired with the two detectors are shown in Fig. 4. The count rate markedly decreased when the depth of the line source in the phantom increased. At lateral position 0 mm, the signal intensity decreased to 37 and 46% for 10-mm depth compared with 0-mm depth for detectors A and B, respectively.

Detector B had better spatial resolution (lower FWHM and FWTM) than detector A at all depths investigated (Table 1). The largest relative difference in FWHM (38%) was seen at the 20-mm depth and the largest relative difference in FWTM (116%) was seen at the 0-mm depth. Furthermore, the spatial resolution worsened with increasing line source depth for both detectors.

**Table 1:** Spatial resolution, given as full-width-at-half-maximum (FWHM) and full-width-at-tenth-maximum (FWTM), for the two detectors when the ^177^Lu line source was placed at different depths in the phantom of tissue-equivalent plastic. For some depths, FWTM was larger than 140 mm and could thus not be determined

	FWHM (mm)		FWTM (mm)	
Depth (mm)	Detector A	Detector B	Detector A	Detector B
0	11	9.9	39	18
10	27	20	88	46
20	41	30	124	77
40	68	49	>140	135
60	93	73	>140	>140
80	113	91	>140	>140

### Organ thickness dependence

**Fig. 5:**
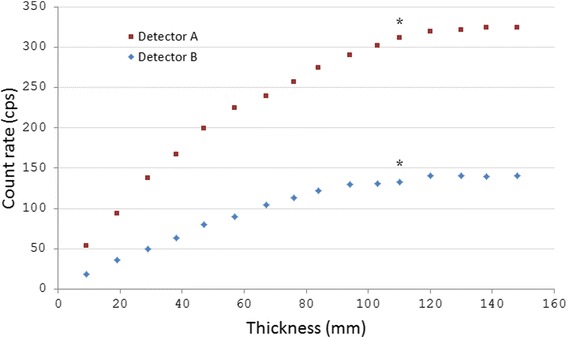
The organ thickness dependence of the two detectors. The count rate was determined for homogeneously distributed ^177^Lu in agarose gel with a thickness of 10–148 mm. Both detectors showed saturation at about 110 mm. The *star* indicates the largest thickness where a statistically significant difference was found compared with the signal intensity at 148 mm

When the thickness of the agarose gel phantom containing radioactivity increased, the count rates of the detectors increased (Fig. 5). For thicknesses larger than about 100 mm, however, a saturation of the signal intensity could be seen. Specifically, the largest thickness where a statistically significant difference was found when the signal intensity was compared with the signal intensity measured at the largest thickness (148 mm) was 110 mm for both detectors.

### Tumour detectability

**Fig. 6:**
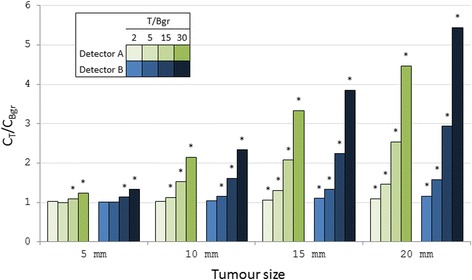
The ratio between the number of counts over the tumour sphere (*C*_*T*_) and that over the background (*C*_Bgr_) vs. the diameter of the tumour sphere, *C*_*T*_/*C*_Bgr_, was generally higher for detector B than for detector A. The *stars* indicate the combinations of T/Bgr and tumour sphere size where a statistically significant difference (*p* < 0.05) between *C*_*T*_ and *C*_Bgr_ could be detected

Generally, the ratio between the number of counts over the tumour sphere and that over the background (*C*_*T*_/*C*_Bgr_) increased both with increasing T/Bgr and size of the tumour sphere (Fig. 6). Furthermore, detector B showed somewhat higher *C*_*T*_/*C*_Bgr_ than detector A. However, the combinations of T/Bgr and tumour sphere size where a statistically significant difference (*p* < 0.05) between *C*_*T*_ and *C*_Bgr_ was found were the same for both detectors. Given the measurement time of 20 s, a statistically significant difference could be seen for all combinations except for the two smallest sizes (5 and 10 mm) for T/Bgr = 2 and for the smallest size (5 mm) for T/Bgr = 5.

The homogeneity (SEM/mean) for the different parts of the agarose gel tumour phantom was 1.8% for the tumour spheres and 2.2% for the background blocks.

## Discussion

The development of a new treatment method where ^177^Lu-octreotate is locally administered to the liver requires quantification of ^177^Lu activity concentration in tumours and normal tissues in vivo. For this therapeutic situation, both the choice of radionuclide (with lower yield of countable photons emitted) and the activity concentration will be much different from the diagnostic situation. Since intraoperative gamma detectors are designed for measurements of diagnostic radionuclides and low amounts of activity, the maximum measurable activity was a parameter of interest in this study. At high activity levels, part of the signal will be lost due to dead-time effects in the detector. This effect can be corrected for if the detector response curve is known. A more significant problem will occur if the activity in a target region is too high to be measured at all. This problem occurred for both detectors. However, detector B proved to be better suited for measurements of high activities compared with detector A, permitting about 5 times higher activity to be quantified. One reason for this was the higher sensitivity of detector A, leading to a higher count rate for a given activity. Another reason was that detector B could reliably measure higher count rates than detector A. Unfortunately, the measurement time of detector B could not be set to less than 10 s. Detector B failed to report the total number of counts during 10 s when the count rate exceeded 9999 cps, due to a limitation in the number of figures of the display window. The maximum count rate of detector A was instead reached at around 6000 cps; higher count rates resulted in an error message, probably due to problems with dead-time effects. The difference in sensitivity also affected the range in which a linear response could be seen, which was almost ten times higher for detector B than for detector A. However, correction for dead-time effects can be done according to the shape of the response curve and thereby yield the actual activity in the tissue of interest. With these results in mind, a low sensitivity may be preferable for intraoperative gamma detector measurements in therapeutic situations, where large amounts of activity or activity concentrations are involved.

The difference in overall sensitivity between the two evaluated detectors was large, 1200 cps/MBq for detector A compared with 500 cps/MBq for detector B. With the two measurable photon energies from ^177^Lu (113 and 208 keV) in mind, the difference in energy window settings of the two detectors are likely to have substantially contributed to the difference in sensitivity. The energy window threshold of 110 keV of detector A means that both ^177^Lu photon energies was measured. The energy window of detector B (112–169 keV), on the other hand, rejected most 208-keV photons. Furthermore, differences in sensitivity could also be explained by collimator geometry. Unfortunately, information on the collimator length of detector A was not available from the manufacturer (Fig. 1). Therefore, comparison of FOV size between the two detectors at different distances was not possible. Lastly, scintillation crystals are often thicker than semiconductor crystals, which was the case for the two detectors evaluated here. Detector A has a crystal thickness of 9 mm (CsI(Tl), effective atomic number, *Z*_eff_ = 54), compared with the 2-mm-thick crystal of detector B (CdTe, *Z*_eff_ = 52), and the detectors had similar *Z*_eff_ values. With a thicker crystal, a larger portion of the incident radiation will interact, and the sensitivity will increase. This also contributes to the higher sensitivity of the scintillation detector (detector A).

Detector B had better spatial resolution than detector A, more apparent for FWTM than for FWHM (Fig. 4). FOV substantially affects spatial resolution, and it is likely that detector B has the smaller FOV. Furthermore, collimator material and thickness are important. Both detectors had very similar collimation, about 3-mm thick tungsten. A thicker collimator would result in better rejection of radiation originating from regions outside the FOV, and thus a better spatial resolution, but also a lower overall sensitivity. Therefore, increasing the collimator wall thickness for determination of ^177^Lu activity concentration in vivo during a therapy situation could be beneficial, both to reduce contribution from adjacent tissues and also to reduce sensitivity. The energy window setting may also influence the spatial resolution. The low-energy threshold of 110 keV of detector A allowed for a larger part of scattered radiation to be detected, compared with the narrower energy window of detector B (112–169 keV). Since scattered radiation generally decreases spatial resolution, this could explain the superior results of detector B.

Overall, the collimation of both detectors was acceptable, with a lateral response of about 5–10% (the count rate at a lateral distance of 20 mm from the line source relative to that at 0 mm from the line source). This can be compared with the results by Benjegård et al., who reported a much higher lateral response, of about 10–40%, for similar types of intraoperative detectors [31]. This large difference could be explained by differences in radionuclides and collimator material used. Benjegård et al. used ^111^In, with higher photon energies (170 and 240 keV) and thus greater penetrability than those of ^177^Lu (113 and 208 keV). Furthermore, the detector with the highest lateral response (40%) in the study by Benjegård et al*.* had a 3-mm-thick lead collimator, while the detectors evaluated in this study had collimators made of tungsten, which has slightly better attenuation properties than those of lead.

During the measurements of organ thickness dependence, the count rate reached saturation at large thicknesses for both detectors. The contribution from the bottom gel slice to the detector signal decreased, both due to increased attenuation by the gel blocks but also due to the increased geometrical distance. Theoretically, the count rate should increase when the thickness increases also at very large thicknesses, but in practice this increase was so small that it was negligible and the signal response curve reached a plateau. Dead-time effects in the detectors could also have contributed to this plateau. To be able to correctly translate a measured count rate from a thick organ into an activity concentration, it is important that the shape of this response curve is known. The results from the measurements of organ thickness dependence could be used for translation of a measured count rate into an activity concentration of ^177^Lu in vivo. However, possible contribution from inhomogeneous activity distribution in the tissue and ^177^Lu in deeper-located tissues should be considered.

The ability of an intraoperative gamma detector to detect a tumour in surrounding normal tissue depends on the size of the tumour, but also on the ratio of the activity concentrations in the two tissues. Therefore, the tumour detectability results were affected by both spatial resolution and overall sensitivity. The C_T_/*C*_Bgr_ values were generally higher for detector B than for detector A, indicating better tumour detectability for detector B, due to the better spatial resolution of detector B. However, statistical analysis resulted in similar performance for both detectors for the tumour sizes and activity concentration ratios evaluated, probably due to the higher sensitivity of detector A. Results from clinical studies where intraoperative gamma detectors were used for radioguided surgery suggest that the lowest *C*_*T*_/*C*_Bgr_ level needed to detect a tumour in surrounding normal tissue is around 1.2–1.5 [18, 21, 22, 43], but higher levels of up to 2 have been suggested as the ratio value corresponding to the lowest limit of detectability [44–47]; the reason for this difference is probably due to the sensitivity of the detector, the number of photons sampled and the biological uncertainties in radionuclide concentration in tissues. Both detectors evaluated in the present work could be used clinically, but the clinical application would decide which parameter is more important, and thus which detector would be best. For low levels of activity concentration, sensitivity may be more important than spatial resolution, and then detector A would be the choice. However, for small structures/regions, such as a small tumour, spatial resolution can be more important than sensitivity, and then, detector B would be better.

To our knowledge, this study is the first evaluation of intraoperative detection using the therapeutic radionuclide ^177^Lu, and the sensitivities measured here were 1200 and 500 cps/MBq. Previous studies have reported sensitivities of 8400 cps/MBq [35], 3800–7300 cps/MBq [30] and 250–2300 cps/MBq [34] for ^99m^Tc and of 2800–6100 cps/MBq for ^111^In [31]. While the sensitivities of the two detectors evaluated in the present study are generally lower than those previously reported, a direct comparison is not completely accurate, since the experimental setup and the choice of radionuclide differed among the studies. Both the emission yield (90.6 and 94.1% for ^111^In, 89.0% for ^99m^Tc and 6.17 and 10.4% for ^177^Lu [39]) and the available energy window settings, as well as the source-detector distance (SDD), will substantially affect the measured sensitivities.

In contrast to sensitivity, spatial resolution is less dependent on the radionuclide. Results from other studies where similar experimental setups were used are summarised in Table 2. Two of these studies included detector B in their evaluations, and these values are presented separately. Overall, the values summarised in Table 2 are in about the same range as those reported in other studies.

**Table 2:** Summary of spatial resolution of intraoperative gamma detectors used in the present study and detectors reported in the literature [27, 30–32, 34, 35]. Zaburlini et al. [34] and Haigh et al. [32] included detector B in their evaluations and their results for this detector is shown separately

	This study		Zamburlini et al. [34]		Haigh et al. [32]		Johnsrud et al. [35]	Benjegård et al. [31]	Britten [30]	Waddington et al. [27]
Radionuclide	^177^Lu		^99m^Tc		^99m^Tc/^111^In/^125^I		^99m^Tc	^111^In	^99m^Tc	^111^In
Number of detectors	2		6		5		1	3	3	2
	FWHM (mm)									
Depth (mm)	Detector A	Detector B	Detector B	All detectors	Detector B	All detectors				
0	11	9.9			35/27/24	12–35/18–28/16–28	20–30^a^	5.2–8.7		
5									11–16	
10	27	20						8.3–23	13–20	
20	41	30						11–35	19–30	12–43
30			35	20–60					26–41	
40	68	49								
50									38–56	23–82
60	93	73								
80	113	91								

^a^Two measurements were made for the same probe, one with and one without an extra collimator

The main focus of this study was to investigate the possibility of determining ^177^Lu activity concentrations in vivo. However, for evaluation of the effect of ionising radiation on tumour and normal tissues, absorbed dose (rather than activity concentration) is the physical quantity of interest. In order to translate activity concentrations to absorbed doses, information about radionuclide biokinetics, absorbed fractions and organ masses are needed [48]. Radionuclide biokinetics could be estimated by repeated measurements of the activity concentration after ^177^Lu-octreotate administration, for example, by an intraoperative detector or a gamma camera.

In summary, sensitivity and spatial resolution are closely related. A good spatial resolution (low FWHM) comes at the expense of a loss in sensitivity, and vice versa. Normally, when the aim is to locate lesions after injection of a low amount of a diagnostic radionuclide, a low sensitivity is undesirable. In the case of a ^177^Lu-octreotate treatment, where there same requirements to minimise the injected amount does not exists, the level of activity is much higher and a high sensitivity is thus less crucial. An intraoperative gamma detector with a good spatial resolution, and a lower sensitivity, is therefore preferable for determination of ^177^Lu activity concentration in vivo.

## Conclusions

The evaluated detectors showed differences in technical performance, especially concerning sensitivity and maximum measureable activity. Still, when the detectors are used for intraoperative measurement of ^177^Lu during therapy we believe that acceptable results will be obtained for both detectors, probably with better results for detector B. The study demonstrates the need to calibrate and evaluate detectors using phantoms simulating the clinical situation and to correct for factors such as depth, organ thickness and dead-time effects to be able to accurately determine ^177^Lu activity concentration in tissues in vivo. Altogether, the results indicate that intraoperative measurements of ^177^Lu distribution in patients may be performed with satisfactory accuracy if accurate corrections for the foregoing factors can be implemented.

## Abbreviations

Cps: Counts per second
FOV: Field-of-view
FWHM: Full-width-at-half-maximum
FWTM: Full-width-at-tenth-maximum
NET: Neuroendocrine tumour
SD: Standard deviation
SDD: Source-detector distance
SEM: Standard error of the mean

## References

[1] Massironi S, Sciola V, Peracchi M, Ciafardini C, Spampatti MP, Conte D (2008). Neuroendocrine tumors of the gastro-entero-pancreatic system. World J Gastroenterol.

[2] Vinik AI, Woltering EA, Warner RR, Caplin M, O'Dorisio TM, Wiseman GA (2010). NANETS consensus guidelines for the diagnosis of neuroendocrine tumor. Pancreas.

[3] Ramage JK, Ahmed A, Ardill J, Bax N, Breen D, Caplin M (2012). Guidelines for the management of gastroenteropancreatic neuroendocrine (including carcinoid) tumours (NETs). Gut.

[4] van Essen M, Krenning EP, de Jong M, Valkema R, Kwekkeboom DJ (2007). Peptide receptor radionuclide therapy with radiolabelled somatostatin analogues in patients with somatostatin receptor positive tumours. Acta Oncol.

[5] Bodei L, Cremonesi M, Ferrari M, Pacifici M, Grana CM, Bartolomei M (2008). Long-term evaluation of renal toxicity after peptide receptor radionuclide therapy with 90Y-DOTATOC and 177Lu-DOTATATE: the role of associated risk factors. Eur J Nucl Med Mol Imaging.

[6] Kwekkeboom DJ, de Herder WW, Kam BL, van Eijck CH, van Essen M, Kooij PP (2008). Treatment with the radiolabeled somatostatin analog [177Lu-DOTA0, Tyr3] octreotate: toxicity, efficacy, and survival. J Clin Oncol.

[7] Gabriel M, Andergassen U, Putzer D, Kroiss A, Waitz D, von Guggenberg E (2010). Individualized peptide-related-radionuclide-therapy concept using different radiolabelled somatostatin analogs in advanced cancer patients. Q J Nucl Med Mol Imaging.

[8] Swärd C, Bernhardt P, Ahlman H, Wängberg B, Forssell-Aronsson E, Larsson M (2010). [177Lu-DOTA0-Tyr3]-octreotate treatment in patients with disseminated gastroenteropancreatic neuroendocrine tumors: the value of measuring absorbed dose to the kidney. World J Surg.

[9] Bodei L, Cremonesi M, Grana CM, Fazio N, Iodice S, Baio SM (2011). Peptide receptor radionuclide therapy with 177Lu-DOTATATE: the IEO phase I-II study. Eur J Nucl Med Mol Imaging.

[10] Claringbold PG, Brayshaw PA, Price RA, Turner JH (2011). Phase II study of radiopeptide 177Lu-octreotate and capecitabine therapy of progressive disseminated neuroendocrine tumours. Eur J Nucl Med Mol Imaging.

[11] Paganelli G, Sansovini M, Ambrosetti A, Severi S, Monti M, Scarpi E (2014). 177Lu-Dota-octreotate radionuclide therapy of advanced gastrointestinal neuroendocrine tumors: results from a phase II study. Eur J Nucl Med Mol Imaging.

[12] Forssell-Aronsson E, Spetz J, Ahlman H (2013). Radionuclide therapy via SSTR: future aspects from experimental animal studies. Neuroendocrinology.

[13] Valkema R, Pauwels SA, Kvols LK, Kwekkeboom DJ, Jamar F, de Jong M (2005). Long-term follow-up of renal function after peptide receptor radiation therapy with 90Y-DOTA0, Tyr3-octreotide and 177Lu-DOTA0, Tyr3-octreotate. J Nucl Med.

[14] Svensson J, Mölne J, Forssell-Aronsson E, Konijnenberg M, Bernhardt P (2012). Nephrotoxicity profiles and threshold dose values for 177Lu-DOTATATE in nude mice. Nucl Med Biol.

[15] Bodei L, Kidd M, Paganelli G, Grana CM, Drozdov I, Cremonesi M (2015). Long-term tolerability of PRRT in 807 patients with neuroendocrine tumours: the value and limitations of clinical factors. Eur J Nucl Med Mol Imaging.

[16] Heller S, Zanzonico P (2011). Nuclear probes and intraoperative gamma cameras. Semin Nucl Med.

[17] Povoski SP, Neff RL, Mojzisik CM, O'Malley DM, Hinkle GH, Hall NC (2009). A comprehensive overview of radioguided surgery using gamma detection probe technology. World J Surg Oncol.

[18] Ahlman H, Tisell L, Wängberg B, Nilsson O, Fjälling M, Forssell-Aronsson E (1994). Somatostatin receptors on neuroendocrine tumors—a way to intraoperative diagnosis and localization. Yale J Biol Med.

[19] Ahlman H, Wängberg B, Tisell L, Nilsson O, Fjälling M, Forssell‐Aronsson E (1994). Clinical efficacy of octreotide scintigraphy in patients with midgut carcinoid tumours and evaluation of intraoperative scintillation detection. Br J Surg.

[20] Wängberg B, Forssell-Aronsson E, Tisell L, Nilsson O, Fjälling M, Ahlman H (1996). Intraoperative detection of somatostatin-receptor-positive neuroendocrine tumours using indium-111-labelled DTPA-D-Phe1-octreotide. Br J Cancer.

[21] Skånberg J, Ahlman H, Benjegård SA, Fjälling M, Forssell-Aronsson E, Hashemi SH (2002). Indium-111-octreotide scintigraphy, intraoperative gamma-detector localisation and somatostatin receptor expression in primary human breast cancer. Breast Cancer Res Treat.

[22] Benjegård SA, Forssell-Aronsson E, Wängberg B, Skånberg J, Nilsson O, Ahlman H (2001). Intraoperative tumour detection using 111In-DTPA-D-Phe1-octreotide and a scintillation detector. Eur J Nucl Med.

[23] Harvey WC, Lancaster JL (1981). Technical and clinical characteristics of a surgical biopsy probe. J Nucl Med.

[24] Szypryt E, Hardy J, Colton C (1986). An improved technique of intra-operative bone scanning. J. Bone Joint Surg. Br. Vol..

[25] Curtet C, Vuillez J, Daniela G, Aillet G, Chetanneau A, Visset J (1990). Feasibility study of radioimmunoguided surgery of colorectal carcinomas using indium-111 CEA-specific monoclonal antibody. Eur J Nucl Med.

[26] Davidson B, Waddington W, Short M, Boulos P (1991). Intraoperative localization of colorectal cancers using radiolabelled monoclonal antibodies. Br J Surg.

[27] Waddington WA, Davidson BR, Todd-Pokropek A, Boulos PB, Short MD (1991). Evaluation of a technique for the intraoperative detection of a radiolabelled monoclonal antibody against colorectal cancer. Eur J Nucl Med.

[28] Millaire A, Hossein‐Foucher C, Rousseau J, Bedoui H, Ducloux G, Marchandise X (1994). A miniature cesium iodide‐photodiode detector for ambulatory monitoring of left ventricular function. Med Phys.

[29] Tiourina T, Arends B, Huysmans D, Rutten H, Lemaire B, Muller S (1998). Evaluation of surgical gamma probes for radioguided sentinel node localisation. Eur J Nucl Med.

[30] Britten AJ (1999). A method to evaluate intra-operative gamma probes for sentinel lymph node localisation. Eur J Nucl Med.

[31] Benjegård SA, Sauret V, Bernhardt P, Wängberg B (1999). Evaluation of three gamma detectors for intraoperative detection of tumors using 111In-labeled radiopharmaceuticals. J Nucl Med.

[32] Haigh PI, Glass EC, Essner R (2000). Accuracy of gamma probes in localizing radioactivity: in-vitro assessment and clinical implications. Cancer Biother Radiopharm.

[33] Kopelman D, Blevis I, Iosilevsky G, Reznik A, Chaikov A, Weiner N (2005). A newly developed intra-operative gamma camera: performance characteristics in a laboratory phantom study. Eur J Nucl Med Mol Imaging.

[34] Zamburlini M, Keymeulen K, Bemelmans M, Brans B, Kemerink GJ (2009). Comparison of sentinel gamma probes for 99mTc breast cancer surgery based on NEMA NU3-2004 standard. Nucl Med Commun.

[35] Johnsrud K, Skretting A, Naum AG, Bogsrud TV, Bach‐Gansmo T (2013). Characterization of an asymmetric add‐on collimator used with a hand‐held gamma probe for radioguided surgery and sentinel node detection: a demonstration of an alternative collimation method. Clin Physiol Funct Imaging.

[36] Todorović-Tirnanić M, Kaemmerer D, Prasad V, Hommann M, Baum RP (2013). Intraoperative somatostatin receptor detection after peptide receptor radionuclide therapy with 177Lu-and 90Y-DOTATOC (Tandem PRRNT) in a patient with a metastatic neuroendocrine tumor. Recent Results Cancer Res.

[37] Collamati F, Pepe A, Bellini F, Bocci V, Chiodi G, Cremonesi M (2015). Toward radioguided surgery with β-decays: uptake of a somatostatin analogue, DOTATOC, in meningioma and high-grade glioma. J Nucl Med.

[38] Collamati F, Bellini F, Bocci V, De Lucia E, Ferri V, Fioroni F (2015). Time evolution of DOTATOC uptake in neuroendocrine tumors in view of a possible application of radioguided surgery with β − decay. J Nucl Med.

[39] Nuclear Decay Data in the MIRD Format. National Nuclear Data Center. 2015. http://www.nndc.bnl.gov/mird/. Accessed 3 Aug 2015.

[40] Forssell-Aronsson E, Bernhardt P, Nilsson O, Tisell L-E, Wängberg B, Ahlman H (2004). Biodistribution data from 100 patients iv injected with 111In-DTPA-D-Phe1-octreotide. Acta Oncol.

[41] Gillams A, Cassoni A, Conway G, Lees W (2005). Radiofrequency ablation of neuroendocrine liver metastases: the Middlesex experience. Abdom Imaging.

[42] Mazzaglia PJ, Berber E, Milas M, Siperstein AE (2007). Laparoscopic radiofrequency ablation of neuroendocrine liver metastases: a 10-year experience evaluating predictors of survival. Surgery.

[43] Bonjer HJ, Bruining HA, Pols HA, de Herder WW, van Eijck CH, Breeman WA (1997). Intraoperative nuclear guidance in benign hyperparathyroidism and parathyroid cancer. Eur J Nucl Med.

[44] Martin EW, Mojzisik CM, Hinkle GH, Sampsel J, Siddiqi MA, Tuttle SE (1988). Radioimmunoguided surgery using monoclonal antibody. Am. J. Surg..

[45] Tuttle SE, Jewell SD, Mojzisir CM, Hinkle GH, Colcher D, Schlom J (1988). Intraoperative radioimmunolocalization of colorectal carcinoma with a hand‐held gamma probe and MAb B72. 3: Comparison of in vivo gamma probe counts with in vitro MAb radiolocalization. Int J Cancer.

[46] Reuter M, Mortz R, de Heer K, Schäfer H, Klapdor R, Desler K (1992). Detection of colorectal carcinomas by intraoperative RIS in addition to preoperative RIS: surgical and immunohistochemical findings. Eur J Nucl Med.

[47] Schirmer WJ, O'Dorisio TM, Schirmer TP, Mojzisik CM, Hinkle GH, Martin EW (1993). Intraoperative localization of neuroendocrine tumors with 125I-TYR(3)-octreotide and a hand-held gamma-detecting probe. Surgery.

[48] Bolch WE, Eckerman KF, Sgouros G, Thomas SR (2009). MIRD pamphlet no. 21: a generalized schema for radiopharmaceutical dosimetry—standardization of nomenclature. J Nucl Med.

